# A causal inference perspective on the analysis of compositional data

**DOI:** 10.1093/ije/dyaa021

**Published:** 2020-03-10

**Authors:** Kellyn F Arnold, Laurie Berrie, Peter W G Tennant, Mark S Gilthorpe

**Affiliations:** d1 Leeds Institute for Data Analytics, University of Leeds, Leeds, UK; d2 School of Medicine, University of Leeds, Leeds, UK; d3 The Alan Turing Institute, London, UK

**Keywords:** Compositional data, collider bias, relative effects, joint effects, causal inference, directed acyclic graphs

## Abstract

**Background:**

Compositional data comprise the parts of some whole, for which all parts sum to that whole. They are prevalent in many epidemiological contexts. Although many of the challenges associated with analysing compositional data have been discussed previously, we do so within a formal causal framework by utilizing directed acyclic graphs (DAGs).

**Methods:**

We depict compositional data using DAGs and identify two distinct effect estimands in the generic case: (i) the total effect, and (ii) the relative effect. We consider each in the context of three specific example scenarios involving compositional data: (1) the relationship between the economically active population and area-level gross domestic product; (2) the relationship between fat consumption and body weight; and (3) the relationship between time spent sedentary and body weight. For each, we consider the distinct interpretation of each effect, and the resulting implications for related analyses.

**Results:**

For scenarios (1) and (2), both the total and relative effects may be identifiable and causally meaningful, depending upon the specific question of interest. For scenario (3), only the relative effect is identifiable. In all scenarios, the relative effect represents a joint effect, and thus requires careful interpretation.

**Conclusions:**

DAGs are useful for considering causal effects for compositional data. In all analyses involving compositional data, researchers should explicitly consider and declare which causal effect is sought and how it should be interpreted.

**Key Messages**

- Directed acyclic graphs (DAGs) provide a useful conceptual tool to consider causal effects for compositional data.
- In the case of compositional data, two distinct causal effect estimands may be of interest—the total (‘unconditional’) effect and the relative (‘collider-conditional’) effect.
- For compositional data with variable totals, both the total and relative effects may be identifiable and causally meaningful, depending upon context. For compositional data with fixed totals, only the relative effect can be identified.
- Where both the total and relative effects are identifiable, researchers must be clear about which effect is being sought and estimated, as the two effects represent distinct (and possibly radically different) quantities with distinct interpretations.

## Introduction

Compositional data comprise the parts of some whole, for which all parts sum to that whole;^1^ the whole itself may vary across units of analysis (e.g. total energy intake) or remain fixed (e.g. total hours in a day). Almost all data are potentially compositional—in the sense that most concepts can be considered part of a greater whole and/or subdivided into smaller parts—though data are often explicitly conceptualized as compositional when there is interest in understanding the role of one or more component(s) in relation to the whole.

Many of the inherent challenges associated with analysing compositional data have been widely discussed,^1–3^ though none have sought to explore these challenges within a formal causal framework by utilizing directed acyclic graphs (DAGs). This is despite the fact that compositional data are commonplace in health and social science research and utilization of DAGs is becoming increasingly widespread due to the insights they provide into historical ‘paradoxes’.^4–6^

In this paper, we use DAGs to consider the causal analysis of compositional data and outline what we believe are the benefits of doing so. We define two distinct effects that may be of interest, and consider their utility and interpretation in the context of three specific example scenarios. Our primary aim is to describe the nuances of identifying and estimating causal effects in the context of compositional data, and to provide a systematic approach to thinking about the specific analytical and interpretational issues that may arise.

## Directed acyclic graphs

DAGs are nonparametric causal diagrams, in which variables are connected by unidirectional arrows. These arrows represent hypothesized direct causal relationships, though do not indicate the magnitude or functional form of such relationships. Two variables may also be connected by indirect causal pathways, which are sequences of arrows that all flow in the same direction and connect the variables through other mediating variables. The only prohibition is that a variable cannot be connected to itself by an indirect causal pathway (i.e. a variable cannot indirectly cause itself).^7^

**Figure 1:**
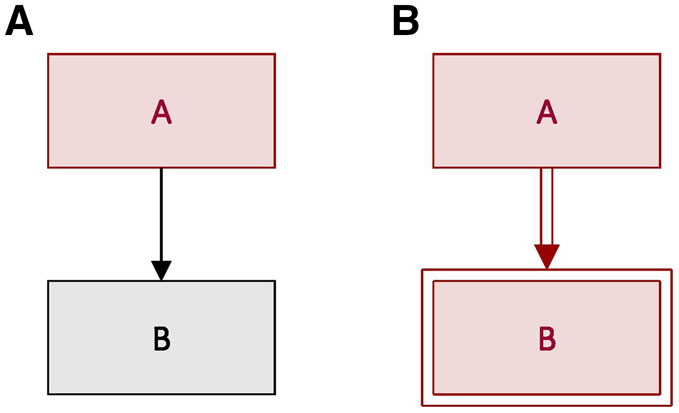
Directed acyclic graphs (DAGs) depicting two random variables *A* and *B*. (A**)** *A* causes *B* probabilistically; this is indicated by a single-lined arrow. (B**)** *A* causes *B* deterministically; this is indicated by a double-lined arrow and double-outlined rectangle.

The causal relationships for which DAGs are typically used are probabilistic in nature. That is, they can be represented by statements like A affects the probability of B (e.g. smoking affects the probability of lung cancer). A simple DAG illustrating this scenario is given in Figure 1A.

However, DAGs may also be used to represent deterministic relationships—i.e. where A fully determines B (e.g. how birthweight determines classification of macrosomia). A simple DAG illustrating this scenario is given in Figure 1B, in which we introduce several notational changes: (i) deterministic relationships are indicated by double-lined arrows; and (ii) fully determined nodes are indicated by double-outlined rectangles.^8^ DAGs in this context are in some sense ‘semiparametric’ because there are parametric constraints implied by the deterministic relationships.

In the next section, we use the framework of DAGs to depict and consider compositional data, which feature deterministic relationships.

## Causal effects for compositional data

**Figure 2:**
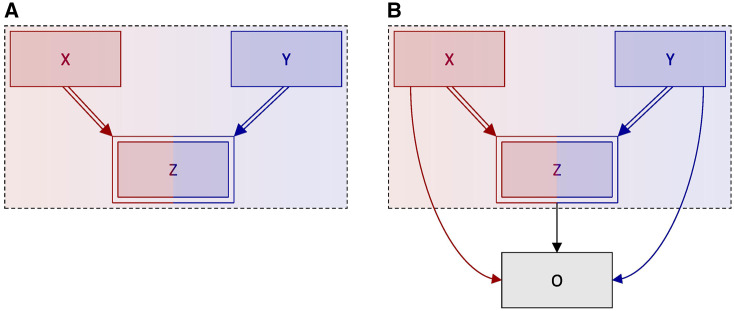
Directed acyclic graphs (DAGs) depicting three random variables *X*, *Y* and *Z*, for which *X + Y = Z*. Deterministic relationships are indicated by double-lined arrows, and fully determined nodes are indicated by double-outlined rectangles. A dashed box around variables indicates that those variables occur at an instantaneous point in time. (A**)** *X* and *Y* are unconditionally independent. (B**)** *X* and *Y* are unconditionally independent, and may affect a subsequent outcome *O* via their influence on *Z*. We note that, due to the deterministic nature of *X*, *Y* and *Z*, it is not possible to parameterize all arrows simultaneously.

Consider three random variables—*X*, *Y* and *Z*—for which *X + Y = Z*. The relationship among these variables is depicted in the DAG in Figure 2A, which employs the previously introduced notation for deterministic relationships. Although *X* and *Y* (the ‘components’) together determine *Z* (the ‘whole’ or ‘total’), no time flow is indicated by the double arcs from the components to the total. Compositional data are unique in that the component parts and the total—which denote the same variable at different levels of aggregation—occur simultaneously. To reinforce this point, we place a dashed box around all compositional variables to indicate they represent the same event in time.

The benefit of depicting compositional data as we have done in Figure 2A is that the causal structure immediately becomes apparent as a ‘collider’ structure.^7^ This structure implies that the components are (unconditionally) independent but become dependent when conditioning on the total (i.e. the ‘collider’). The reason for this is simple—conditioning on *Z* may be thought of as ‘filtering’ by *Z* or holding *Z* constant; therefore, any change in one of the components (*X* or *Y*) must be accompanied by an equal and opposite change in the other.^7^ For example, in the absence of conditioning on *Z*, increasing *X* by one unit also increases *Z* by one unit, but crucially does not affect *Y*. In contrast, increasing *X* by one unit while holding *Z* constant means *Y* must decrease by one unit.

The dependency that arises between *X* and *Y* when conditioning on *Z* has implications for causal analyses. Suppose we consider *X*, *Y* and *Z* in relation to a subsequent outcome *O* (Figure 2B). In the absence of conditioning on *Z*, changing either *X* or *Y* can be thought to affect changes in *O* by changing *Z*. However, conditioning on *Z* blocks these indirect paths, such that changes in *X* or *Y* must affect changes in *O* directly. This indicates the existence of two distinct effects for the effect of each component on the outcome.

Without loss of generality, suppose we are interested in the causal effect of the component *X* on the outcome *O*. The two effects are:

- The total (‘unconditional’) effect of *X* on *O*: this estimand captures the effect on *O* of increasing *X* (and thereby increasing *Z*), regardless of *Y*. [Note that we refer to this effect as ‘unconditional’ because it represents the effect in which the total *Z* is not conditioned upon; it does not imply that no other conditioning (e.g. for confounders) may be made.]
- The relative (‘collider-conditional’) effect of *X* on *O*: this estimand captures the effect on *O* of increasing *X* while simultaneously decreasing *Y*. [Note that the identifiability conditions^9^ for the relative effect of *X* on *O* may be stronger than those for the total effect of *X* on *O*. We have omitted confounders from consideration for simplicity of illustration, but it is theoretically possible that confounders of the *X*–*O* relationship differ from those of the *Y*–*O* relationship. In such a scenario, the relative effect would require conditioning on the confounders of both the *X*–*O* and *Y*–*O* relationships, whereas the total effect would require conditioning only on the confounders of the *X*–*O* relationship.]

In the setting of compositional data, in which *Z* is fully determined by its component parts, both effects may be of interest depending upon the context; this is contrary to perceived wisdom in the generic (i.e. probabilistic) case, in which conditioning on a collider is considered to be undesirable. Indeed, the dependency induced between two independent events when conditioning on a common descendant is often referred to as ‘collider bias’ as it has the potential to cause serious interpretational problems for causal analyses (see, e.g. the ‘birthweight paradox’^10^).

In the following sections, we discuss the previously defined total and relative effects in the context of several example scenarios involving compositional data, and the resulting implications for causal analyses involving data of this kind. We note that in certain situations these effects may not be considered sufficiently ‘well-defined’, since they do not correspond to unique interventions and therefore represent unknown combinations of all possible exposure mechanisms.^11^ Our focus, however, is not on debating the validity of causal inference in the absence of well-defined interventions, but on demonstrating the conceptual issues that arise in the analysis of compositional data.

## Compositional data with variable totals

We first consider causal inference for compositional data with variable totals, which are compositional data for which the ‘total’ can vary across units of analysis. Examples include:

- total height (decomposed into leg length and trunk length);
- total fat mass (decomposed into brown fat mass and white fat mass);
- total population (decomposed into 0–18, 19*–*35, 36*–*60 and >61 year-olds).

We consider the total and relative effects for two specific example scenarios, and the resulting implications for compositional data with variable totals.

### Scenario 1: economically active population and gross domestic product

**Figure 3:**
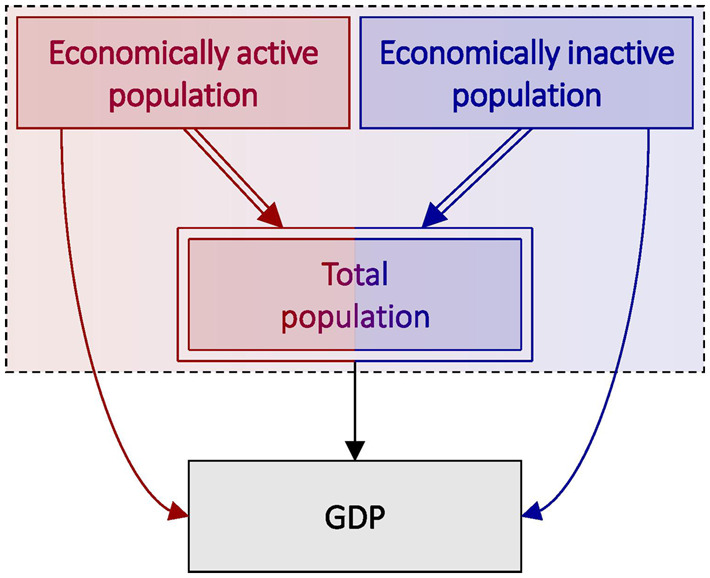
Directed acyclic graph (DAG) depicting total population in relation to gross domestic product (GDP), in which total population is subdivided into economic activity and inactivity (i.e. total population = economically active population + economically inactive population). Deterministic relationships are indicated by double-lined arrows, and fully determined nodes are indicated by double-outlined rectangles. A dashed box around variables indicates that those variables occur at an instantaneous point in time.

Suppose we are interested in the causal effect of the total number of economically active individuals within a geographical area on the area-level gross domestic product (GDP). The DAG in Figure 3 represents this scenario, which also explicitly depicts the compositional nature of the exposure (i.e. economically active population + economically inactive population = total population); confounders are omitted for ease of illustration. In this scenario, both total and relative effects of the economically active population on GDP are obtainable, and both may have utility depending on the context.

The total effect of the economically active population represents the average change in GDP that results from adding economically active individuals to the area, thereby increasing both the number of economically active individuals and the total number of individuals, while doing nothing to the population of economically inactive individuals. An estimate of this effect may be of interest if, e.g. the government were considering policies aimed at increasing economic immigration.

In contrast, the relative effect of the economically active population represents the average change in GDP achieved by swapping economically inactive individuals for economically active individuals—either by adding economically active individuals and removing an equal number of economically inactive individuals, or by effectively converting economically inactive individuals into economically active individuals (or some combination thereof). The relative effect is therefore a joint effect—it is the combined effect of simultaneously increasing the economically active population while decreasing the economically inactive population by equal numbers, thereby retaining the same total population. An estimate of this effect may be of interest if, e.g. the government were considering job-training programmes for currently unemployed individuals.

In this scenario, both the total and relative effects reflect the population-level average effects of changing the relative numbers (i.e. the proportions) of economically active individuals to alter GDP, but by different means. We may therefore derive two distinct causal quantities, each of which may be of interest depending on the context or hypothetical intervention.

### Scenario 2: fat consumption and body weight

**Figure 4:**
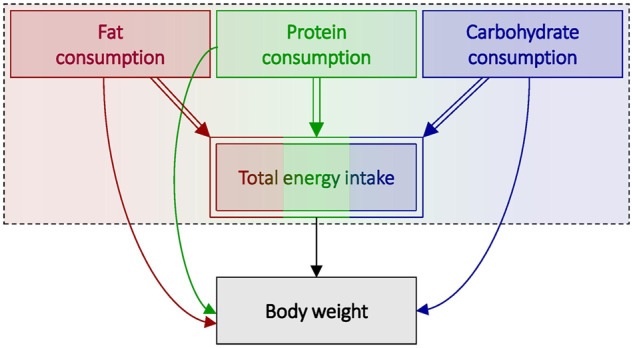
Directed acyclic graph (DAG) depicting total energy intake in relation to body weight, in which total energy intake is subdivided into macronutrient consumption (i.e. total energy intake = fat consumption + protein consumption + carbohydrate consumption*)*. Deterministic relationships are indicated by double-lined arrows, and fully determined nodes are indicated by double-outlined rectangles. A dashed box around variables indicates that those variables occur at an instantaneous point in time.

Now, suppose we are interested in the causal effect of fat consumption on body weight. The DAG in Figure 4 represents this scenario, which also explicitly depicts the compositional nature of diet (i.e. fat consumption + protein consumption + carbohydrate consumption = total energy intake).

The total effect of fat consumption represents the average change in weight that results from adding fat to an individual’s diet, irrespective of the consumption for all other macronutrients, which consequently increases total energy intake without altering other consumption behaviours. An estimate of this effect may be of interest if, e.g. individuals were considering a diet that advocated reducing and/or eliminating fat and not replacing it with other macronutrients (e.g. by reducing or eliminating cooking oil).

The relative effect of fat consumption represents the average change in weight that results from replacing all ‘other’ macronutrient consumption (i.e. protein and carbohydrate consumption, in their relative proportions) with fat consumption such that fat consumption is increased without increasing total energy. This is again a joint effect that incorporates both the effects of increasing fat consumption and reducing the consumption of other macronutrients. An estimate of this effect may be of interest if, e.g. individuals were considering a diet that advocated replacing fat from their diet with ‘other’ macronutrients (e.g. replacing high-fat foods with their lower-fat counterparts).

Similar to scenario 1, each effect captures a different approach to increasing the relative amount of fat intake, and each may yield radically different estimates according to different contextual interpretations. Whereas each causal effect may arguably have a meaningful interpretation, each must be considered carefully, and its interpretation made explicit according to the context sought.

### Implications

For analyses involving compositional data with variable totals, both the total and relative effects of a particular component may be identifiable and interpretable, depending upon context. However, care must be taken when referring to the relative causal effect of one component, as in reality the estimate captures the joint effect of this component and all other components that have not been conditioned upon.

In the instance that only two components are considered (e.g. scenario 1), conditioning on the total uses one degree of freedom, meaning that the two components share only one degree of freedom and thus represent just one single (binary) variable (i.e. economically active and not economically active). In such a scenario, the relative effect of the component of interest is unavoidably interconnected with the effect of the other component; it represents the effect of replacing the first component with the second, which is equal and opposite to the effect of replacing the second component with the first. The causal effect of each component only has meaning relative to the other, and therefore they are fundamentally a single joint effect.

Where three or more components are considered (e.g. scenario 2), this means that the relative effect represents the influence of one component in relation to the average influence of all other components that have not been conditioned upon. Whether this reference provides a meaningful comparison is largely subjective and will depend strongly on context. More specific comparisons can be achieved by conditioning on additional components, thereby restricting the number of components comprising the joint effect. Where the relative effect is estimated, the contribution of all unconditioned reference components should be carefully considered.

## Compositional data with fixed totals

Next, we consider compositional data with fixed totals, which are compositional data for which the ‘total’ is fixed to the same value for every unit of analysis. These types of data usually involve some standard unit of measurement (e.g. time or space) that is fixed by nature or convention. Examples include:

- hours per week (decomposed into time spent commuting, time spent working, time spent sleeping, and ‘other’);
- Boeing 747 capacity (decomposed into adult passengers, child passengers and vacant seats);
- child benefit block grant (decomposed into money spent directly on the child and money not directly spent on the child).

We consider one specific example scenario and discuss the resulting implications for compositional data with fixed totals.

### Scenario 3: time spent sedentary and body weight

**Figure 5:**
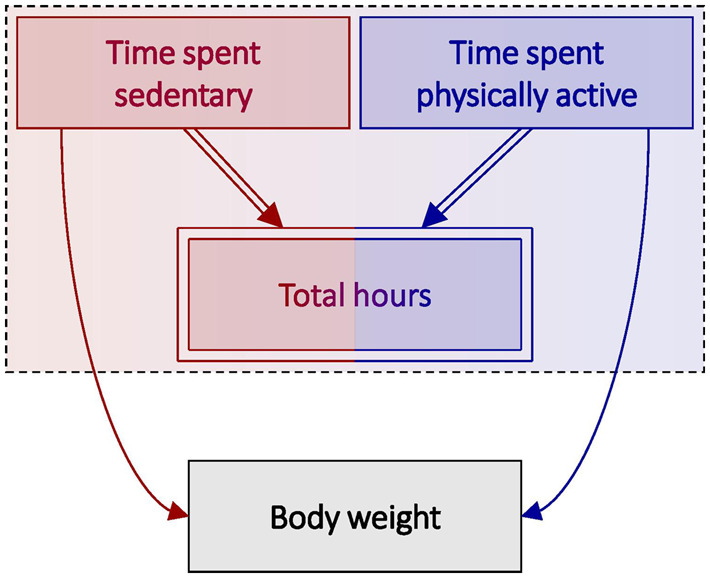
Directed acyclic graph (DAG) depicting total hours in relation to body weight, in which total hours is subdivided into activity category (i.e. total hours = time spent sedentary + time spent physically active). Deterministic relationships are indicated by double-lined arrows, and fully determined nodes are indicated by double-outlined rectangles. A dashed box around variables indicates that those variables occur at an instantaneous point in time. Total hours is inherently constrained (i.e. total hours = 24) and thus has no identifiable causal effect on body weight.

Imagine we are interested in the causal effect of time spent sedentary (i.e. not moving, including sleeping, sitting and standing) per day on body weight. Because total hours per day is fixed at 24 for every individual, there is an inherent constraint imposed upon time spent sedentary and time spent physically active (i.e. time spent sedentary + time spent physically active = 24 h). It is nevertheless useful to consider this constraint—length of day—within a causal framework, since it helps to illustrate many of the same challenges. The DAG in Figure 5 describes this scenario, with confounders omitted for ease of illustration.

Total hours is depicted as a deterministic function of time spent sedentary and time spent physically active. What differentiates this scenario from those considered previously (i.e. in Figures 2–4) is that the total cannot vary, and thus we have depicted it as having no identifiable causal effect. Nevertheless, explicitly including total hours in the DAG is useful for demonstrating the structural constraints that exist for compositional data with fixed totals, and how this implies the existence of relative effects only.

It is impossible to identify or estimate the causal effect on weight of time spent sedentary without also considering the effect of time spent physically active (i.e. the total effect), since any increase in one must be accompanied by an equal and opposite decrease in the other. The constraint imposed by the fixed length of a day demands that the components be considered jointly. Thus, the relative effect is the only effect that can possibly be obtained.

### Implications

For analyses involving compositional data with fixed totals, only the relative causal effect of a particular component is identifiable. The inherent constraint upon a fixed total operates in a similar fashion to conditioning on a variable total, and results in an estimate that represents the effect of one component relative to all other components omitted from the analysis.

In the instance that only two components are considered (e.g. scenario 3), the total constraint (i.e. exactly 24 h in a day for everyone) means that the two components share one degree of freedom and are therefore implicitly a single binary variable (i.e. time spent sedentary and time spent not sedentary). It makes little sense to even conceptualize the two components as having separate effects, since each variable may only be defined and estimated relative to the other. This is important for discussions regarding the relative merits of decreasing one component vs increasing another (e.g. decreasing sedentary behaviour vs increasing physical activity^12–14^), as the two are not distinct entities from a causal perspective. Where more than two components are considered (e.g. where time spent physically active is further subdivided into light, moderate and vigorous exercise), care should be taken to select the most meaningful and/or appropriate joint effect.

## Conclusion

The analysis of compositional data is challenging from a causal inference perspective, where conditioning on the total (a ‘collider’) creates a dependency between the components. This dependency does not preclude meaningful causal interpretation, but it does require careful consideration of the joint nature of causal effects in such situations.

Where only two components exist, as in scenario 1, the meaning of the joint (or relative) effect is straightforward, as it represents the effect of swapping one component for the other. However, where more than two components exist, as in scenario 2, the joint effect instead represents the effect of swapping one component for a combination of the other components. In such situations, the total effect likely represents a more important estimand, although conditioning on additional component(s) can be implemented to identify more specific substitution effects.

Inherent constraints on the total are also present for some situations involving compositional data, as in scenario 3; such constraints function similarly to conditioning and restrict interpretation. The relative effects that characterize data of this type are well-recognised in other contexts. For instance, categorical data may be conceptualized as a trivial case of compositional data, in which the total is fixed at one. Indeed, this notion is implicit in the coding of such variables for statistical analysis—each category is treated as a binary variable with value zero or one, and the sum of all categories for every individual equals one (i.e. each individual may belong to one and only one category). In such situations, one category must be specified as the reference category and all other effect estimates must be interpreted relative to this category. These issues are also recognised in the context of age–period–cohort analyses, where data are tabularized into intervals such that three concepts are perceived with only two degrees of freedom (i.e. age + cohort = period).^15^

In all situations involving compositional data, it is paramount that researchers explicitly consider and declare which causal effect is sought and how it should be interpreted, since the total and relative effects have the potential to be radically different, even if both are causally meaningful. For example, the effect on cardiovascular disease of eating red meat on top of an otherwise healthy diet may be drastically different to the effect of replacing ‘healthy’ dietary components with red meat. Insufficient clarity regarding the distinction between these two effects likely contributes to ongoing confusion due to apparently contradictory results.^16^^,^^17^ Across all contexts, careful attention must be paid to recognising these issues and reporting results consistent with the analyses undertaken.

## Funding

This work was supported by the Economic and Social Research Council [grant number ES/J500215/1 to K.F.A.], the Medical Research Council [grant number MR/K501402/1 to L.B.] and The Alan Turing Institute [EP/N510129/1 to P.W.G.T. and M.S.G.].

**Conflict of interest: **None declared.
